# A High Accuracy Time-Reversal Based WiFi Indoor Localization Approach with a Single Antenna †

**DOI:** 10.3390/s18103437

**Published:** 2018-10-12

**Authors:** Lili Zheng, Binjie Hu, Haoxiang Chen

**Affiliations:** Guangdong Provincial Key Laboratory of Short-Range Wireless Detection and Communication, School of Electronic and Information Engineering, South China University of Technology, Guangzhou 510640, China; zll_scut@163.com (L.Z.); chenhaoxiang.scut@gmail.com (H.C.)

**Keywords:** time reversal, Channel State Information, indoor localization, clustering

## Abstract

In this paper, we study the influence of multipath magnitude, bandwidth, and communication link number on the performance of the existing time-reversal (TR) based fingerprinting localization approach and find that the localization accuracy deteriorates with a limited bandwidth. To improve the localization performance, by exploiting two unique location-specified signatures extracted from Channel State Information (CSI), we propose a high accuracy TR fingerprint localization approach, HATRFLA. Furthermore, we employ a density-based spatial clustering algorithm to minimize the storage space of the fingerprint database by adaptively selecting the optimal number of fingerprints for each location. Experimental results confirm that the proposed approach can efficiently mitigate accuracy deterioration caused by a limited bandwidth and consequently, achieve higher accuracy compared with the existing TR localization approach.

## 1. Introduction

With the rapid development of communication technology, more and more electronic equipment is being connected to the Internet, which has led to a rise in innovative applications such as smart homes, smart cities, and so on. However, accurately obtaining the positions of the terminals is critical to the promotion of the above applications. The Global Position System (GPS), as a mature positioning system, has became a widely deployed and useful tool for our lives. Though it can offer meter-level position information outside, GPS is not suitable for indoor localization since obstacles such as concrete and furniture in indoor environments block the relatively weak GPS signals, thus resulting in incorrect positioning. Currently, several indoor localization methods based on different infrastructures have been proposed: the Wireless Fidelity (WiFi) based method, the infrared based method, the wireless sensor based method and so on. Among them, WiFi based indoor localization methods have attracted extensive attention due to the widespread deployment of WiFi devices. In the WiFi based indoor localization methods, the Received Signal Strength (RSS) and the Channel State Information (CSI) [1] are two essential variables/indexes for positioning. Therefore, there are two mainstream WiFi based indoor localization methods: the RSS-based method and the CSI-based method. Specifically, in the RSS-based method, the fingerprinting scheme [2,3,4,5] has the advantages of good real-time capacity and low requirements on the equipment compared to the model-based scheme [6].

Compared with the RSS-based method, the CSI-based one can achieve higher precision in indoor localization. This is because CSI not only contains signal strength information, but also contains phase information [7]. Furthermore, the CSI above each sub-carrier can be measured by leveraging the property of Orthogonal Frequency Division Multiplexing (OFDM).

To alleviate the small-scale fading effect, a model based method FILA [8] weights the average CSI over 30 sub-carriers to obtain the effective CSI. Besides, based on the free space path loss propagation model, a refined indoor propagation model was proposed to represent the relationship between the effective CSI and the distance. Experiments verified that the effective CSI is more stable than RSS, and the localization performance of CSI-based approach outperforms the RSS-based one. As a reference, the Cramer-Rao Lower Bound on CSI-based localization error is derived in [9].

Some Angle of Arrival (AoA) estimation based methods have been proposed. SpotFi [10] uses the CSI of three receiver antennas to build a smoothed CSI matrix. Through eigenvalue decomposition of the covariance matrix, the high resolution noise subspace is obtained and thus, the AoA and Time to Flight (ToF) of the signal can be jointly estimated. In this way, sub-meter level localization precision can be achieved. WIPP [11] reconstructs the CSI matrix to guarantee that the number of direction measurement units is larger than the number of signal paths and uses the affinity propagation clustering algorithm to identify the direct signal path from the target to each Access Point (AP). PILA [12] uses two-dimensional spatial smoothing to rebuild the CSI matrix and verifies its effectiveness by AoA estimation. In addition, to locate the target, the Second-Order Cone Programming relaxation approach is used to transform the localization problem into a convex one and location estimation of the target can be obtained based on the least squares criterion.

A Time Difference of Arrival (TDOA) based indoor localization system, ToneTrack, Ref. [13] improves the localization accuracy by increasing the available bandwidth based on channel switches. Considering the direct path may be totally blocked, triangle inequality and clustering schemes have been proposed to remove the invalid APs. Due to channel attenuation and hardware imperfection, the collected CSIs are likely to be distorted. To improve the resolution of the derived power delay profile, Splicer [14] splices the CSI measurements from multiple WiFi frequency bands, wherein a set of key techniques has been proposed to separate the mixed hardware errors from the collected CSI measurements. Given that the available frequency bands of WiFi are discontinuous, Chronos [15] adopts the inverse Non-uniform Discrete Fourier Transform to compute sub-nanosecond ToF by using the measurements over these discontinuous frequency bands.

Besides, CSI can be used to further improve fingerprinting localization’s precision. The authors in [16] quantified the power of a package by using CSI and utilized the summational CSI as fingerprints. Their experimental results showed that, compared with RSS, using CSI for fingerprint localization significantly enhanced the localization accuracy. FIFS [17] leverages the CSI values including different amplitudes and phases at multiple propagation paths, known as the frequency diversity, to uniquely manifest a location and adopts a probabilistic model to determine the location of a target. To reduce the fingerprint size, CSI-MIMO [18] subtracts the amplitude and phase values from subsequent sub-carriers and uses the difference in mean of the amplitude and phase as the fingerprints. In DeepFi [19], a deep learning based indoor fingerprinting localization system is proposed. In the offline stage, the amplitude of CSI is used to train the Deep Belief Networks and the weights of the deep network are chosen as fingerprints. In the online stage, a probabilistic method based on the Radial Basis Function is employed to determine the location of the target. Similarly, PhaseFi [20] uses both the amplitude and transformed phase of CSI and BiLoc [21] uses the amplitude of CSI and estimated AoA as the input data for deep networks, respectively.

Based on the existing literature, it is clear that, in indoor environments, the precision of the aforementioned localization methods is affected by the existence of multipaths in indoor environments to some extent. In contrast, Time-Reversal (TR) based indoor localization methods utilize the fact that signals received at different locations go through different communication paths caused by the multipath to distinguish one location from the other. When the TR received signal is passed back, energy is focused onto the intended location, which is called the spatial focusing effect [22]. By utilizing the location-specific characteristic of multipaths, a TR indoor localization system (TRIPS) was proposed in [23]. Specifically, in this method, in the offline phase, a database is created by mapping the physical geographical location with logical location in the Channel Impulse Response (CIR) space. In the online phase, the location of the target is determined by matching the estimated CIR with those in database. Note that in TRIPS, only a single antenna is needed, while sub-decimeter-level precision can be achieved. In our previous work [24], we conducted a preliminary study of the factors influencing TR localization. It is known that CSI can be obtained by transforming CIR from the time domain to the frequency domain, and the convolution in the time domain is equivalent to the inner product in the frequency domain. The authors in [25] calculated the resonating strength in the frequency domain with a total concatenated bandwidth of 1GHz and realized a perfect 5 cm localization precision. Considering the bandwidth limit in the mainstream WiFi system, a frequency hopping approach named WiFi-TRIPS was proposed in [26]. In this method, when calculating the resonating strength in WiFi-TRIPS, the measured CSI over multiple continuous/discontinuous channels is employed. It was shown in [27] that not only the frequency diversity but also the spatial diversity can be utilized to improve the localization precision. Considering that, the spatial diversity is determined by the number of communication links and the frequency diversity is determined by the number of available channels. In general, for a TR based fingerprinting localization system, a larger concatenated bandwidth and more communication links are helpful to improve the localization accuracy.

Though TR based fingerprinting localization methods are able to achieve sub-decimeter level precision which is better than other methods, there is still much work to be done. The existing TR based fingerprinting localization methods mainly take the matching degree of CSI amplitude into account, while CSI contains not only the amplitude information but also phase information. As a result, we find that the performance of the existing TR based fingerprinting localization method deteriorates with the limited bandwidth. Besides, for each location, it is hard to determine the number of fingerprints to be stored in database.

To address these issues, we propose a high accuracy TR fingerprinting localization approach called HATRFLA to improve the localization accuracy. The contributions of our work include the following:

- We respectively study the influence of different factors on TR fingerprinting localization’s performance; we conduct three experiments and propose an improved metric to quantize these influences.
- In the offline stage of HATRFLA, a density-based clustering algorithm is used to adaptively obtain the number of fingerprints to be stored for each location. To our knowledge, this is first time that the density-based clustering algorithm has been used to optimize the fingerprint selection.
- In the online stage of HATRFLA, both the amplitude and phase of CSI are jointly considered. Based on this, two unique location-specified signatures are extracted and used to determine the location of the target. Thus, a higher localization accuracy can be achieved. As far as we know, this is the first time that a location-specified signatures based on the phase of CSI has been introduced into TR based localization.
- To highlight the proposal but without loss of generality, in our experiments, we only consider the simplest experimental setting, i.e., only a single communication link with a single 20 MHz channel under Non-Light Of Sight (NLOS) can be measured to obtain the CSI, which is common in life, but can be seen as a challenge for high accuracy localization. The experimental results show that the proposed algorithm performs well even in this case.

The structure of this article is as follows. In Section 2, we give the architecture description and describe the proposed HATRFLA in the offline stage and the online stage, respectively, in detail. The experimental results are given and discussed in Section 3, followed by the conclusions in Section 4.

## 2. A High Accuracy TR Based Fingerprinting Localization Approach

**Figure 1:**
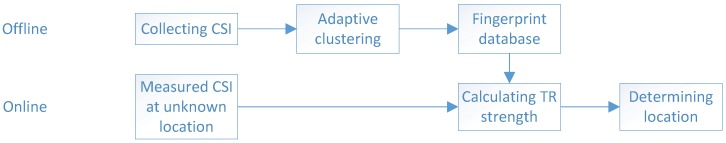
Architecture description.

The architecture description of the proposed approach is shown in Figure 1. There are two main stages: an offline stage and an online stage. In the offline stage, CSI is measured at the receiver. A density-based clustering algorithm is used to remove measurement noise and determine the number of clusters. Finally, for each cluster, the amplitude and phase of each center member are stored as fingerprints in a database. In this way, the number of fingerprints that needs to be stored for each location can be adaptively determined. In the online stage, a linear transformation is used to obtain the location-specific signature of the CSI phase information. In addition, an improved resonating strength of TR jointly considering both the amplitude and the transformed phase is calculated for localization. Each step mentioned above is explained in detail in the following sub-sections.

### 2.1. Offline Stage

#### 2.1.1. CSI Collection

Standard 802.11n [28] gives the definition of CSI based on the Multiple-Input Multiple-Output Orthogonal Frequency Division Multiplex (MIMO-OFDM) system and uses it as a part of a feedback mechanism to improve communication links via beamforming. CSI describes the channel properties of each communication link between the transmitter and receiver over each data sub-carrier. The CSI measurement of a packet includes two parts, i.e., amplitude and phase:

(1) $$H=\left|H\right|e^{jsin∠H}$$

where |H| is the amplitude and ∠H is the phase. In this paper, we use the CSI which is measured over only a single communication link. It is known as a low equipment requirement for deployment but is challenging for localization. The collected CSI of multiple packets are recorded as

(2) $$H_{N\times M}={\left[H_{1},H_{2},\ldots ,H_{N}\right]}^{T},$$

where *M* is the number of sub-carriers and *N* is the number of packets.

#### 2.1.2. Density-Based Clustering

**Figure 2:**
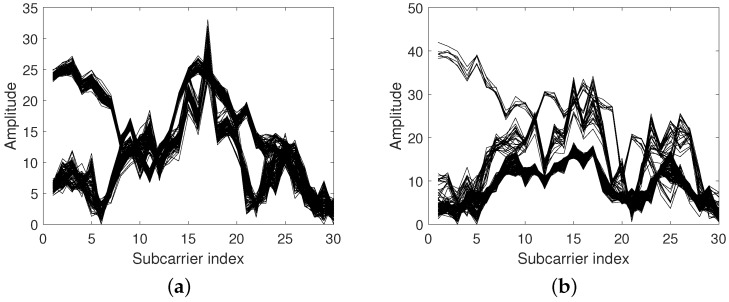
Different cluster numbers for the Channel State Information (CSI) amplitude can be observed at differant locations. (**a**) Amplitude of CSI at location a. (**b**) Amplitude of CSI at location b.

Figure 2 shows the CSI amplitude of 200 consecutive packets measured at two different locations. Two and three emerging clusters can be observed respectively for these two locations, and data belonging to the same cluster seem relatively stable. Reference [29] considered the phenomenon of clusters as being a result of fading which is caused by different electro-magnetic propagation effects and/or environmental changes. By comparing Figure 2a with Figure 2b, it can be inferred that the number of clusters may be different for different locations and is hard to predict for any location. Intuitively, to minimize the number of fingerprints in the database, for each location, we need to adaptively select the number of fingerprints, i.e., the number of clusters, to be stored in the database. Common methods such as K-Means [30] or the Gaussian mixture model based method [29] can be used to set the number of clusters before clustering, and different number selections may result in different clustering results. If we plan to select the cluster members which are the nearest to the center of each cluster as fingerprints at one location, inappropriate cluster number setting, whether larger or smaller, will cause the selected fingerprints to be unable to describe the features of that location correctly. To tackle this issue, we employed the density-based spatial clustering of applications with noise (DBSCAN) algorithm [31] to adaptively obtain the number of clusters. Note that DBSCAN can remove the measurement noise adaptively in the process of clustering. To our knowledge, this is the first time that the density-based clustering algorithm has been used to optimize fingerprint selection. The details of DBSCAN are shown in Algorithm 1.

**Algorithm 1** DBSCAN: Density-based spatial clustering of applications with noise**Require:**$|H_{N\times M}|$: amplitude of H; MinPts: minimum neighbor number requirement for a central point of a cluster; Eps: neighborhood radius;**Ensure:** clustering result *C* 1: $Visit_{N\times 1}={\left[0,0,\ldots ,.0\right]}^{T}$: mark all points in |H| as unvisited points, $Noise_{N\times 1}={\left[0,0,\ldots ,.0\right]}^{T}$: mark all points in |H| as non-noise points, $IDX_{N\times 1}={\left[0,0,\ldots ,.0\right]}^{T}$: mark all points in |H| as the state of not adding any clusters, C=0: initial number of cluster; 2: Normalize |H| 3: **for** each point *p* in |H|
**do** 4:   **if**
Visit(p)=0
**then** 5:    Visit(p)=1; 6:    Calculate the Euclidean distance between this point and the other points and get a set of neighbors N1 which have a distance of less than Eps; 7:    **if**
num(N1)<MinPts
**then** 8:     Noise(p)=1; 9:    **else**10:     C=C+1;11:     k=1;12:     **repeat**13:      k=k+1; IDX(k)=C;14:      **if**
Visit(k)=0
**then**15:       Visit(k)=1;16:       Calculate the Euclidean distance between this point and the other points and get a set of neighbors N2 which have a distance of less than Eps;17:       **if**
num(N2)>=MinPts
**then**18:        N1=N1∪N2; ;19:       **end if**20:      **end if**21:      **if**
IDX(k)=0
**then**22:       IDX(k)=C23:      **end if**24:     **until** k>num(N1)25:    **end if**26:   **end if**27: **end for**

**Figure 3:**
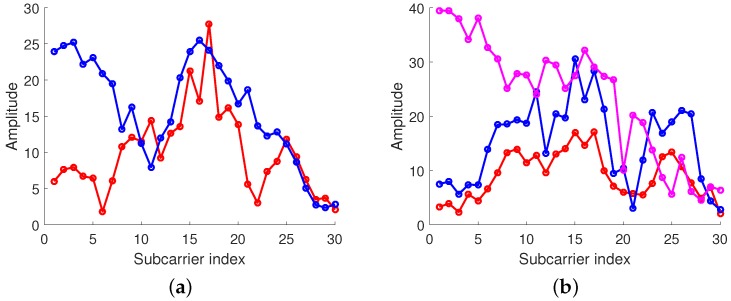
Fingerprints at differant locations. (**a**) Fingerprints at location a. (**b**) Fingerprints at location b.

After clustering, as shown in Figure 3, for each cluster, the cluster member that is nearest to the cluster center is selected as the fingerprint. At this point, the fingerprint set $\hat{D}$ can be obtained at location *d* and expressed as

(3) $$\hat{D}={\left[H_{1}^{\prime },H_{2}^{\prime },\ldots ,H_{D}^{\prime }\right]}^{T},$$

where the subscript *D* represents the number of clusters.

### 2.2. Online Stage

#### 2.2.1. Spatial-Temporal Focusing of TR

**Figure 4:**
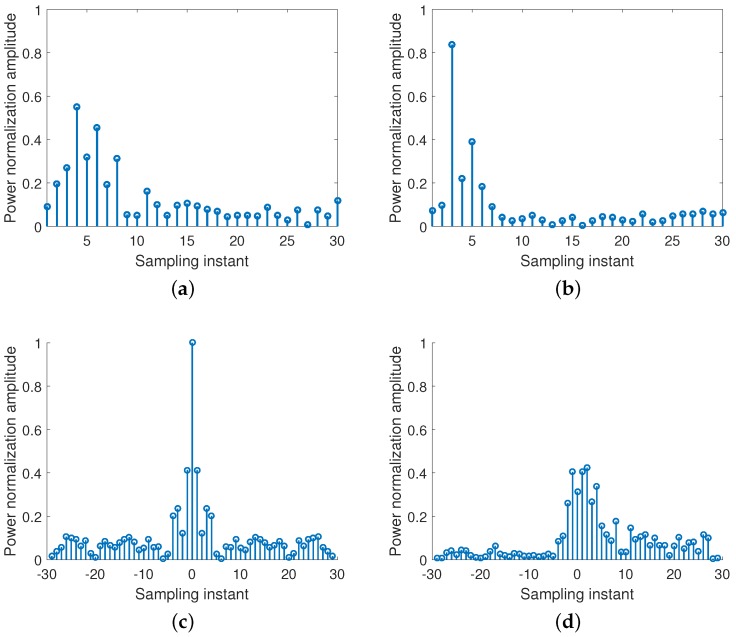
Focusing effect of Time-Reversal (TR). (**a**) Channel Impulse Response at location A. (**b**) Channel Impulse Response at location B. (**c**) Received TR signal at location A. (**d**) Received TR signal at location B.

Assuming the channel is reciprocal, when convolving the sample CIR data with the time-reversed CIR storing in the fingerprint database, only that at the intended location will produce a peak, which is known as spatial-temporal focusing effect [23]. As shown in Figure 4, we randomly selected two locations with an interval of 10 cm, A and B, and then we collected the CIR at these two locations. The distance from these two locations to the receiver was about 4 m. To simplify this problem, but without loss of generality, two locations where the number of CSI clusters was only one were chosen. In addition, after measuring the CSI from location A to the receiver, we obtained the CIR from the CSI by Inverse Fast Fourier Transform (IFFT) and retransmitted the time-reversal CIR to location A and location B. When convolving the retransmitted signal with the CIR from the receiver to location A, we observed an obvious sharp peak at a particular time instant, which is known as the spatial-temporal focusing effect. However, when convolving the retransmitted signal with the CIR from the receiver to location B, the phenomenon is not observed.

By fully utilizing the location-specific spatial-temporal focusing effect, the TR resonating strength can be calculated as follows [23]:

(4) $$\eta^{T}\left(h_{1},h_{2}\right)=\frac{\max\left|\left(h_{1}*g_{2}\right)\left[i\right]\right|}{\sqrt{\sum_{i}^{L-1}{\left|h_{1}\left[i\right]\right|}^{2}}\sqrt{\sum_{i}^{L-1}{\left|g_{2}\left[i\right]\right|}^{2}}}$$

where *L* is the number of usable sub-carriers, $h_{1}$ is the sample CIR data, $h_{2}$ is the fingerprint CIR data stored in the database, and $g_{2}$ is the time-reversed and conjugated version of the fingerprint data $h_{2}$.

Note that the calculation of IFFT may introduce error. Since the convolution in the time domain is equivalent to the inner product in the frequency domain, Equation (4) can be transformed from the time domain to the frequency domain in a CSI-based TR approach [27]. In this case, the resonating strength can be directly calculated with CSI measurement without transformation:

(5) $$\eta^{F}\left[H,H^{\prime }\right]=\frac{\gamma }{\sqrt{\Lambda }\sqrt{\Lambda^{\prime }}}$$

with

(6) $$\gamma =\max_{\phi }\left|\sum_{i=1}^{L}H_{i}H_{i}^{\prime *}e^{\frac{-2\pi j(i-1)\phi }{L}}\right|,$$

(7) $$\Lambda =\sum_{i=1}^{L}{\left|H_{i}\right|}^{2},\;\Lambda^{\prime }=\sum_{i=1}^{L}{\left|H_{i}^{\prime }\right|}^{2}$$

where *H* and $H^{\prime }$ are the sample CSI data and fingerprint CSI data, respectively; Λ and $\Lambda^{\prime }$ are the channel energies of *H* and $H^{\prime }$, respectively; γ is the modified cross-correlation between *H* and $H^{\prime }$; the subscript *i* is the sub-carrier index; the additional phase rotation of $e^{\frac{-2\pi j(i-1)\phi }{L}}$ can be treated as the phase compensation due to synchronization errors; and the value of ϕ ranges from 1 to *L* [27]. Under ideal conditions, the phases cancel each other out and thus, the matching rating of the amplitude is calculated finally.

For each location, the TR resonating strength is firstly calculated between the sample data *H* and all the fingerprints $H_{d}^{\prime }$ stored in fingerprint database *D*, and then, the resonating strength $\eta_{d}^{F}$ can be defined as the maximal value of them at location *d*:

(8) $$\eta_{d}^{F}=\max_{H_{d}^{\prime }\in D}\eta^{F}\left(H,H_{d}^{\prime }\right).$$

Note that the value of $\eta_{d}^{F}$ ranges from 0 to 1, and we can view it as the possibility that one location is correctly recognized as the target location.

In addition, to evaluate the influence of different factors, such as the multipath magnitude in environment, the number of communication links, and the concatenated bandwidth, on the TR fingerprinting localization performance, we propose a spatial-temporal focusing effect metric. The experiment results show that the higher the metric is, the higher the system localization accuracy is. See Section 3.1 for more details.

#### 2.2.2. An Improved Resonating Strength

When there are enough multipath numbers in the environment are present, having available communication links and an efficient concatenated bandwidth are helpful to improve the performance of TR-based fingerprinting localization; this, in turn, raises the requirements for the system equipment. For an area of interest where there is a target that needs to be located, the magnitude of the multipath in the environment is relatively stable. For a common 802.11n system, the mainstream bandwidth is 20 MHz/40 MHz. While TR based localization may not perform well when the CSI measured in a single 20 MHz channel is used (see Section 3.1.3 for details), it is noteworthy that WiFi devices generally work in unauthorized frequency bands where they are susceptible to the same frequency interferences. Therefore, splicing a large efficient bandwidth is not practical due to time cost and equipment requirements. Similar problems exist when multiple communication links are adopted.

To mitigate the performance deterioration and improve performance when there is only a single available communication link with limited bandwidth, a high accuracy TR fingerprinting localization approach, HATRFLA, is proposed in this paper. The key is to note that CSI contains both amplitude and phase information. All the existing TR based localization approaches only treat the amplitude of CSI as a location-specific signature [23] and then use the matching degree between real-time measurements and fingerprints for localization. Beyond that, we notice that the processed phase can also be regarded as another location-specific signature [29]. Instinctively, a higher accuracy localization would be achieved by making full use of the amplitude information and the phase information of CSI.

- Phase Processing

Taking into consideration the hardware imperfections of the system, the phase of CSI is distorted. The raw measured phase $\hat{\psi_{i}}$ for the subcarrier ith can be expressed as [29]

(9) $$\hat{\psi_{i}}=\psi_{i}-2\pi \frac{k_{i}}{N}\delta +\beta +Z$$

where $\psi_{i}$ represents the true phase, δ is the time lag due to the Sampling Frequency Offset (SFO), *N* is the Fast Fourier Transform size, β is the unknown phase, and *Z* is the small measurement noise which can be ignored. However, it is hard to obtain the exact values of δ and β. Here, we adopt a simple linear transformation to remove the effects of δ and β approximately:

(10) $$k=\frac{\hat{\psi_{L}}-\hat{\psi_{1}}}{L-1}$$

(11) $$b=\frac{1}{L}\sum_{i=1}^{L}\psi_{i}.$$

The processed phase can be obtained with

(12) $$\tilde{\psi_{i}}=\hat{\psi_{i}}-ki-b$$

**Figure 5:**
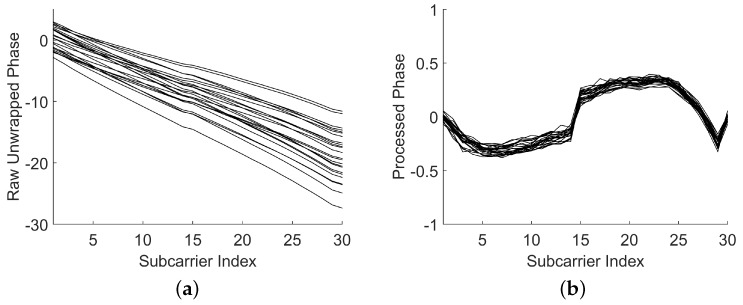
Obtaining a stable phase with 24 packets. (**a**) Raw unwrapped phase. (**b**) Processed phase.

Figure 5 illustrates the raw unwrapped phase collected in the same location and the corresponding processed phase. We can see that the raw unwrapped phase is random; however, the phases after processing are rather stable among different packets. In addition, the processed phase was proven to be a location-specified signature in [29]; hence, we can utilize it to distinguish one location from another. As a reference, the upper bound on the variance of processed phase was proven in [20]. Based on that, we introduce the processed phase as a new evaluating indicator into the proposed approach. It is important to note that it does not require additional fingerprint database space.

- Matching Rating Calculation of the Processed Phase

According to Equation (5), the calculation of the TR resonating strength is approximately equal to the calculation of the cross-correlation between the real-time sample data and the fingerprint data. Since the processed phase is an unique location-specified signature, we can calculate the cross-correction coefficient between two processed phases to evaluate their matching rating. Thus, we can obtain an improved resonating strength through multiplying the calculated cross-correction coefficient by Equation (5).

Specifically, the calculation of the matching rating of processed phase can be given as follows:

(13) $$\mu \left[H,H^{\prime }\right]=\left|\frac{∠\tilde{H}\times ∠\tilde{H^{\prime }}}{\sum_{i=1}^{L}{\left|∠\tilde{H_{i}}\right|}^{2}\sum_{i=1}^{L}{\left|∠\tilde{H_{i}^{\prime }}\right|}^{2}}\right|$$

(14) $$\mu_{d}=\max_{H_{d}^{\prime }\in \hat{D}}\eta \left(H,H_{d}^{\prime }\right)$$

where $∠\tilde{H}$ is the processed phase of ∠H, μ represents the maximal phase matching rating between the sample data $∠\tilde{H}$ with all fingerprints $∠\tilde{H^{\prime }}$ in the database $\hat{D}$. Hence, for each location *i*, the improved resonating strength $\nu_{d}^{proposed}$ can be defined as

(15) $$\nu_{d}^{proposed}=\eta_{d}^{F}\times \mu_{d}.$$

#### 2.2.3. Localization Estimation

Based on the aforementioned analysis, the estimated location $\hat{p}$ can be obtained by maximizing ν among all possible locations:

(16) $$\hat{p}=\arg\max_{p}\nu_{p}^{proposed}.$$

## 3. Experiment and Results

**Figure 6:**
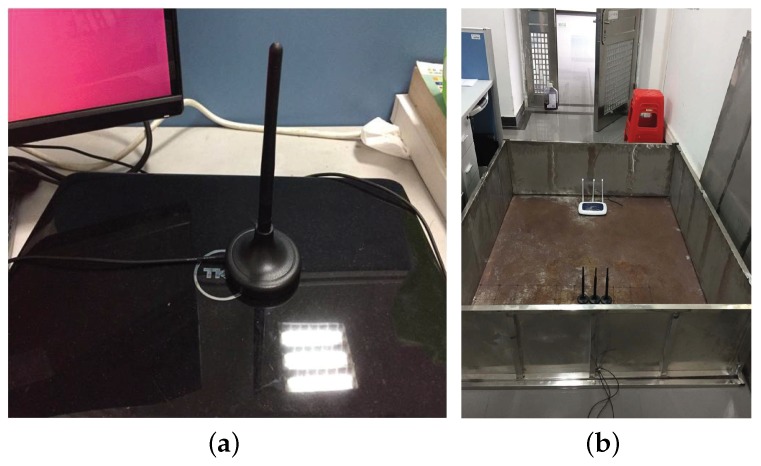
Experimental equipment. (**a**) A DELL laptop equipped with Intel 5300 NIC. (**b**) Metal box.

In this section, we first discuss the influence of factors on the existing TR based localization [27], and then we compare the proposed HATRFLA with the existing TR based localization (see the latest version in [27]). In the experiments, we used a DELL laptop equipped with Intel 5300 NIC as the receiver (see Figure 6a) and a TP-Link router as the transmitter, where Linux CSI tool [1] was adopted to record the CSI over 30 sub-carriers.

### 3.1. Evaluation of the Factors Influencing the Peformance of the Existing TR Based Localization

In this subsection, we discuss the influence of the multipath magnitude in the environment, the communication link number and the bandwidth on the existing TR based localization approach. Furthermore, a metric is proposed to quantify the influence.

#### 3.1.1. Time Reversal Metric

Some metrics [32,33] have been proposed to calculate the spatial-temporal focusing gain of TR in the radio electromagnetic propagation, but they cannot be applied directly in TR based fingerprint localization due to the different available parameters. To better evaluate the influence of these factors on the existing TR based fingerprinting localization, we propose an improved spatial-temporal focusing metric (ISTFM) in the following text. The calculation of the ISTFM is as follows:

(17) $$\tau_{i}=\frac{\eta_{F}^{i}}{\left(\sum_{r=1}^{nn}\eta_{F}^{neighbor_{i,r}}\right)/n_{n}}$$

where $\tau_{i}$ is the proposed spatial-temporal focusing metric at location *i*, $\eta_{F}^{i}$ is the resonating strength at location *i*, $\eta_{F}^{neighbor_{i,r}}$ is the resonating strength at the *r*th neighbor location of location *i*, and $n_{n}$ is the number of neighbor locations. In this paper, we define the locations which are 10 cm away from location *i* as its neighbor locations.

#### 3.1.2. Influence of the Multipath Magnitude on TR Based Localization

In this sub-section, we investigate the influence of the multipath magnitude in the environment on the localization performance. Compared to the case of the typical indoor environment, signal transmission inside a metal box would follow much richer paths from the transmitter to the receiver. We respectively tested the performance at nine locations of a typical indoor environment in a rather rich multipath environment, wherein we used a metal box to create a rich multipath environment (see Figure 6b). In this experiment, these nine locations were arranged with 3 rows × 3 columns when facing the router, and the adjacent test locations were separated by 10 cm. For each test point, the CSI was collected on channel 3 with 20 MHz bandwidth.

**Figure 7:**
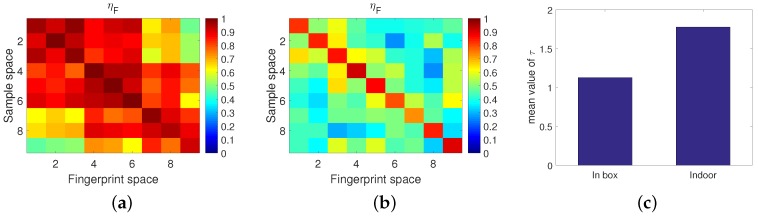
Influence of the multipath component on performance. (**a**) Indoors. (**b**) In the box. (**c**) Spatial-temporal focusing metric comparison.

As shown in Figure 7, the resonating strength was calculated between each sample and the fingerprints of each locations. Under ideal conditions, the values of diagonal elements are maximal since they correspond to the correct location. Compared with some obscure regions in Figure 7a, the contrast between the diagonal elements and the off-diagonal elements in Figure 7b can be observed more clearly. In other words, higher accuracy localization results were achieved. Correspondingly, the ISTFM in a metal box, shown in Figure 7c, is 56.52% higher than that in the typical indoor environment.

In total, compared with other fingerprinting approaches which are affected by the multipath in the environment, we found that using a rich multipath in the environment is helpful to improve the performance of TR based approaches, which makes TR based fingerprints approaches different and even precedes other fingerprinting approaches.

#### 3.1.3. Influence of Bandwidth on TR Based Localization

In accordance with 802.11n specifications [28], the CSI was measured every 2/4 sub-carriers respectively with a 20/40 MHz bandwidth. Taking advantage of the frequency diversity, we used the CSI concatenated from multiple channels to improve the localization accuracy. Since the calculation of the resonating strength was discrete in the frequency domain in this experiment, we spliced the CSI measured into two non-overlapped 20 MHz channels, channel 1 and channel 13, to get the CSI measurements with an efficient bandwidth vof 40 MHz and spliced the CSI measured in two non-overlapped 40 MHz channels, channel 1 and channel 13, to get the CSI measurements with an efficient bandwidth of 80 MHz. We tested the performance in the typical indoor environment at nine locations where adjacent test locations were separated by 10 cm with different bandwidths in the 2.4 GHz frequency band. These nine locations were arranged with 3 rows × 3 columns when facing the router.

**Figure 8:**
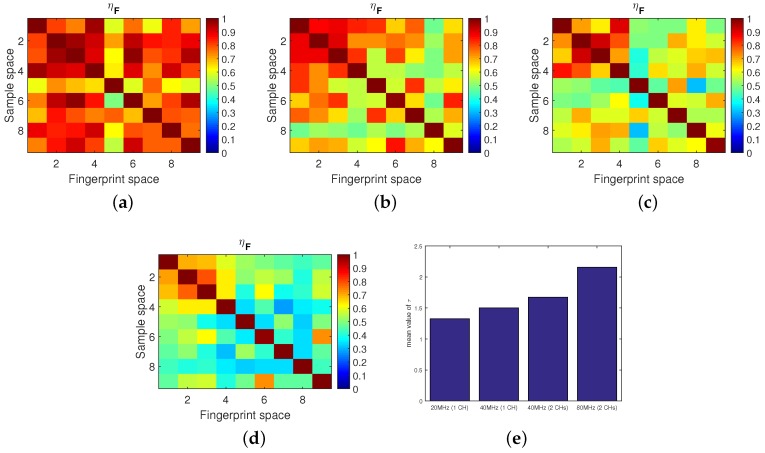
Influence of bandwidth on performance. (**a**) 20 MHz (1 CH). (**b**) 40 MHz (1 CH). (**c**) 40 MHz (2 CHs). (**d**) 80 MHz (2 CHs). (**e**) Spatial-temporal focusing metric comparison.

Note that the measurement with a 20 MHz bandwidth channel included information from 30 sub-carriers, the measurement of a 40 MHz channel included information from 30 sub-carriers, the measurement of two non-overlapped 20 MHz channels included information from 60 sub-carriers, and the measurement of two non-overlapped 40 MHz channel included information from 60 sub-carriers. The localization results are shown in Figure 8; thereinto, CH is the abbreviation of “channel”.

In total, with an increase of bandwidth, the contrast between the diagonal elements and the off-diagonal elements was clearer. Thus, we can conclude that the localization accuracy of TR based approaches can be improved by increasing the bandwidth. Similar results can be observed in Figure 8e, in which the ISTFM with 80 MHz bandwidth is higher than the others.

A special group of comparative experiments were carried out. Though the bandwidth was the same, compared with the performance when using one 40 MHz channel (see Figure 8c), the performance when using two non-overlapped 20 MHz channels (see Figure 8b) was better since the measurement of the latter contains more sub-carrier information, which helps to distinguish one location from another.

It was found that with a bandwidth of 40 MHz or larger, the localization accuracy was rather high, even almost 100%, which generally meets the requirements of localization accuracy. However, when the bandwidth was limited, as Figure 8a shows, the performance of the TR based approaches deteriorated sharply. It is worth noting that, in practice, a bandwidth of 20 MHz is common and mainstream. In addition, measuring the CSI over multiple channels requires the transmitter and receiver to be able to switch the channel simultaneously. This would correspondingly increase the system’s equipment cost and time cost. In the following, we mainly focus on how to improve the localization accuracy with the limited 20 MHz bandwidth.

#### 3.1.4. Influence of the Communication Link Number on TR Based Localization

With the large-scale deployment of the MIMO communication system, we the CSI measured in multiple communication links contains more information than that in a single communication link, i.e., multiple communication links are helpful for distinguishing one location from another in the fingerprinting localization. We tested the performance in the typical indoor environment at nine locations where adjacent test locations were separated by 10 cm with different communication links in the 2.4 GHz frequency band. These nine locations were arranged with 3 rows × 3 columns when facing the router. The adopted bandwidth was set to be 40 MHz.

**Figure 9:**
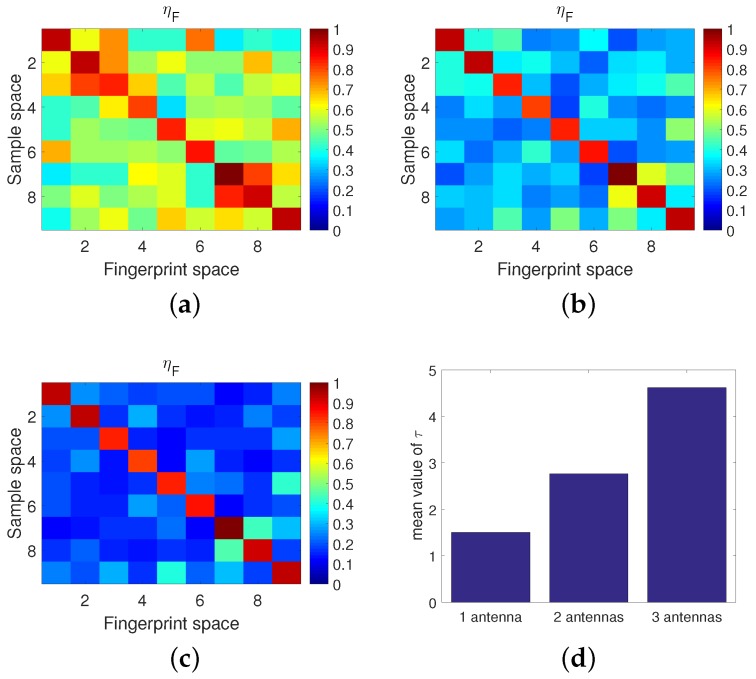
Influence of the communication link number on performance. (**a**) one receiving antenna. (**b**) two receiving antennas. (**c**) three receiving antennas. (**d**) Spatial-temporal focusing metric comparison.

Figure 9 confirms that with an increase in the communication link number, namely, by taking advantage of the space diversity, the contrast between the diagonal elements and the off-diagonal elements is clearer and thus, the localization accuracy of TR based approaches can be improved. Similar results can be observed in Figure 9d, in which the ISTFM with three antennas is higher than the others, and for any one additional antenna, the ISTFM increases by about 75%. It is worth mentioning that a similar conclusion can be achieved by using multiple APs in the TR based fingerprinting localization approach since we can treat multiple APs as another form of multiple communication links. To highlight the high accuracy of the proposed approach, in our paper, only a limited bandwidth (20 MHz) and a single communication link were needed for localization, which is known to be a low requirement for equipment and deployment, although it makes localization hard.

Signals from different locations undergo different reflecting paths and delays to the receiver and thus, cause different amplitudes and phases of CSI which can be regarded as two location-specific signatures; therefore, the existance of adequates multipath in indoor environments makes TR based localization approaches reasonable and suitable. On the other hand, CSI describes the channel state at the sub-carrier level and shows the frequency selecting fading effect on the whole channel. Through concatenating the CSI measured in multiple communication links or multiple channels, more information can be included in the measurements. In this case, the frequency selecting fading effect can be observed more obviously, which helps to distinguish one location from another. To sum up, TR based localization approaches perform well when there are enough multipaths in the environment, a sufficiently large, efficient bandwidth, and/or sufficient communication link numbers.

### 3.2. Evaluation of the Proposed HATRFLA

**Figure 10:**
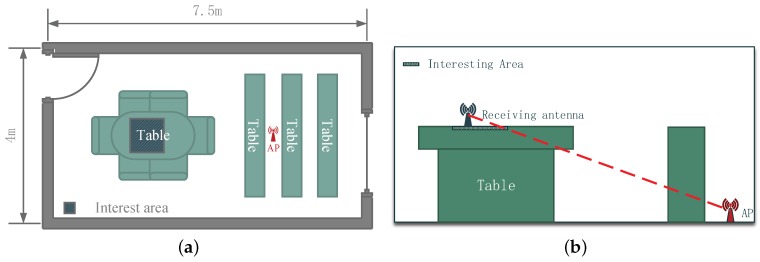
Layout of the meeting room for localization. (**a**) Horizontal direction. (**b**) Vertical direction.

In this part, we compare the performance of the proposed HATRFLA with that of the existing TR based fingerprinting localization system presented in [27] which is known as the latest version and the original TR localization system presented in [23]. The experiment was conducted in a meeting room (see Figure 10a). We evaluated their performance at 36 locations in an area of interest, where adjacent locations were separated by 10 cm. These 36 locations were arranged with 6 rows × 6 columns when facing the router. In this experiment, as shown in Figure 10b, the router was placed on the ground and the receiving antenna was placed on the table. Under such circumstances, the LOS between the transmitter and the receiver is blocked by the furniture; hence, the communication is NLOS. Note that NLOS communication is common and sometimes unavoidable in typical indoor environments, but it increases the difficulty of localization. The number of used communication links was just 1, i.e., the usable antenna at sender was 1 and the usable antenna at the receiver was 1. We adopted 1 AP for localization and the CSI was measured over 1 channel with 20 MHz.

The equipments used in this sub-section were the same as Section 3.1. For each location, the collected 350 CSI packets were divided into two equal-size sets. In HATRFLA, the data in the first set were trained to adaptively obtain fingerprints based on DBSCAN. For competitor 1 [27] and competitor 2 [23], a certain number (ranging from 1 to 20) of data in the first set were randomly chosen and used as fingerprints. In both approaches, the data in the second set were randomly picked up as the sample data to test the localization accuracy.

#### 3.2.1. Adaptive Fingerprint Collection

In the offline stage of HATRFLA, an adaptive fingerprint collection was adopted. As mentioned earlier, the number of clusters of CSI may be different at different locations. After the adaptive fingerprint collection, different numbers of fingerprints can be obtained for different location; while measurement noise is removed automatically.

**Figure 11:**
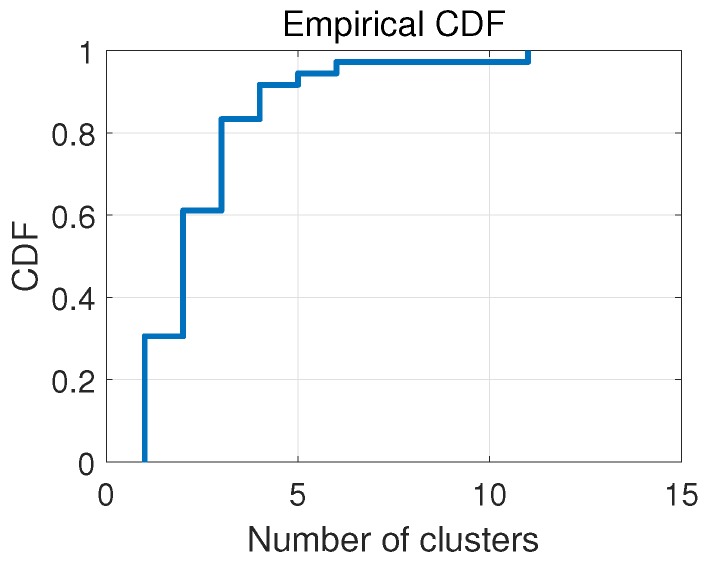
Clustering results at 36 different locations.

Figure 11 shows the Cumulative Distribution Function (CDF) of the fingerprint number at all 36 locations. We can see that the number of clusters in the indoor environment was from 1 to 11, and in most cases, the number of clusters was less than 3. To sum up, the total fingerprint number for these 36 locations was 92 and the average fingerprint number per location was 2.6.

#### 3.2.2. Evaluation of Improved Resonating Strength

**Figure 12:**
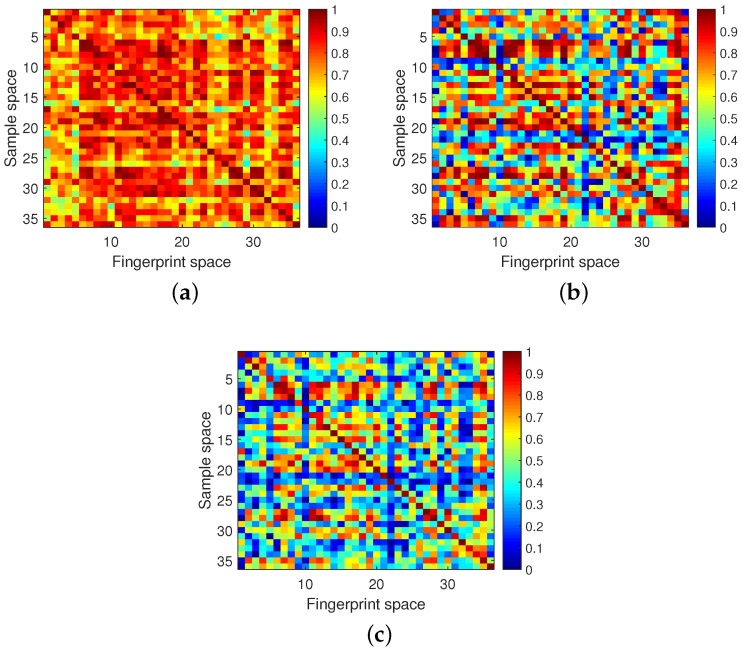
The localization results by using three different parameters. (**a**) $\eta_{d}^{F}$. (**b**) $\mu_{d}$. (**c**) $\nu_{d}^{proposed}$.

In the online stage of HATRFLA, the improved resonating strength at all locations was calculated and the location with the maximal value was supposed to be where the target was. According to Equation (15), $\nu_{d}$ is the product of $\eta_{d}^{F}$ and $\mu_{d}$. To evaluate the effectiveness of the improved resonating strength, the localization results from using the three parameters are given in Figure 12. Different color blocks represent the value of the used parameter(see the color bar on the right side of figure for details). We found that when just $\eta_{d}^{F}$ was used for localization, the resonating strength of these 36 locations ranged from 0.5 to 1 and there many obscure regions existed which led to localization errors. When just $\mu_{d}$ was used for localization, the resonating strength of these 36 locations ranged from 0.1 to 1 and the contrast between diagonal elements and the off-diagonal elements was more clearer than the former. Some obscure regions in former were removed but some new localization errors also emerged. Note that the localization errors shown in Figure 12a are different from those in Figure 12b. Therefore, the complementary relationship between these two results can be used to improve the localization accuracy. The final localization result when using $\nu_{d}^{proposed}$ is shown in Figure 12c; obscure regions were reduced obviously and the localization errors seen in the first two figures were removed. Hence, the validity of the improved resonating strength can be testified.

#### 3.2.3. Comparison of Localization Performance

**Figure 13:**
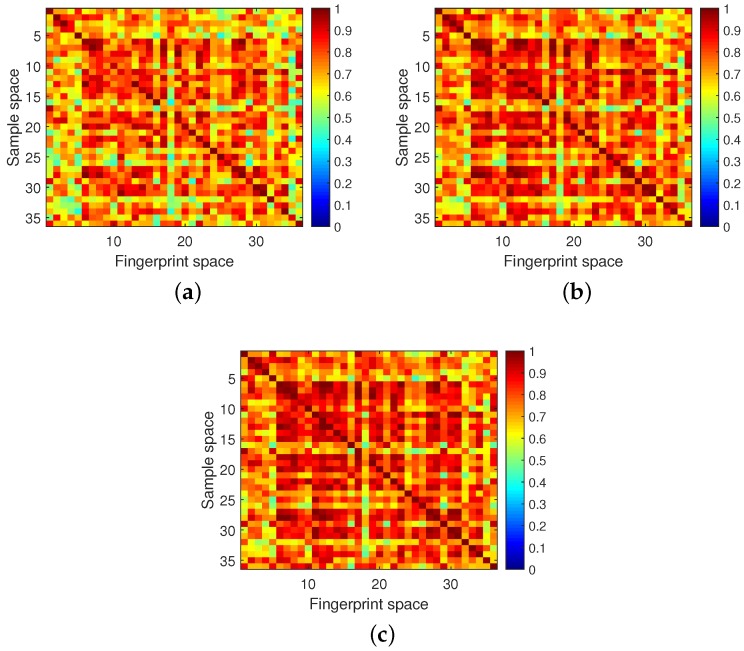
Competitor 1’s localization results. (**a**) One fingerprint for each location. (**b**) Four fingerprints for each location. (**c**) 10 fingerprints for each location.

For comparison, the localization results of competitor 1 [27] are also given at the beginning of this sub-section. We tested the localization performance with different fingerprints for each location. As Figure 13 shows, as the number of fingerprints per location increased, the main diagonal element value increased significantly while, unfortunately, the obscure regions also expanded. In addition, the fingerprints for each location were randomly picked and inappropriate fingerprints resulted in a localization error. Due to the limited 20 MHz bandwidth, even if 10 fingerprints were stored for each location, 100% localization accuracy still could not be achieved. Different from the competitor in [27], the proposal was able to pick fingerprints adaptively for each location. The advantage of the proposal is that it can minimize the number of fingerprints and enable these fingerprints to effectively describe location features.

To further evaluate the effectiveness of the proposed approach, for these 36 locations, we tested each location 10 times and got the corresponding localization error statistics for the proposal, competitor 1 [27], and competitor 2 [23]. For the proposal, once the fingerprints had been adaptively picked, the number of fingerprints remained the same. For the competitors, we changed the number of fingerprints at each location and evaluated the localization performance for each case. The evaluation indexes included the localization accuracy, the mean localization error, and the standard deviation (SD) of the localization error. Particulary, we defined the difference between the true location of the target and the estimated location as the localization error and we obtained the mean localization error by averaging these localization errors in experiments repeated 10 times.

**Figure 14:**
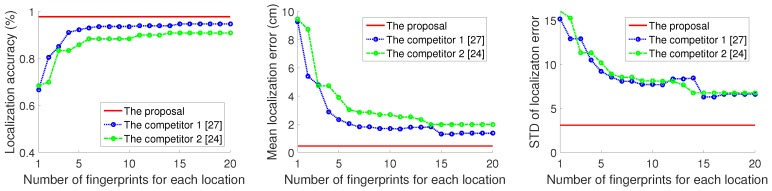
Comparison of localization performance.

The statistical data are shown in Figure 14. The highest accuracy levels produced by competitor 1 and competitor 2 respectively were 94.72% and 90.83%, both of which are less than 100%. The performance of competitor 2 was slightly worse than that of competitor 1, which we think may be caused by the inaccuracy of IFFT in competitor 2. In general, as the number of fingerprints increased, for the competitors, the localization accuracy increased; meanwhile, the mean localization error and SD of the errors decreased. The one exception to the general trend in these results was that when the number of fingerprints at each location was between 13 and 15, the localization accuracy of the competitor 1 remained the same as the number increased, but the mean localization error and SD of the localization error increased. This is because, in competitor, fingerprints are randomly selected which may make the error a bigger one; hence, the increase in fingerprints in this range enlarges the previous localization errors instead of correcting them. Figure 14 demonstrates the disadvantages of random fingerprint selection.

In contrast, though the proposal picked a total of 92 fingerprints for all 36 locations (average 2.6 fingerprints per location), it was always able to achieve higher localization accuracy and lower localization error than the competitors. This is because in the proposed method, the fingerprints are selected adaptively, that is, the number of fingerprints corresponding to each location is different, which greatly reducesthe needed space to store these fingerprints, meanwhile keeping the valid location feature. Besides, by considering the two unique, location-specified signatures, the localization accuracy can be further improved.

In order to further compare the proposal with the competitors, their computation time complexities are discussed. Let *N* be the number of locations. In competitor 1, according to Equation (5)–(8), the computation time complexity of competitor 1 is O(N). In competitor 2, according to Equation (4), the computation time complexity is O(N). In the offline stage of the proposal, assume the number of packets for clustering is *M*, according to the Algorithm 1, the computation time complexity of DBSCAN is O(M×log(M)). Considering clustering is conducted in the offline stage, the corresponding computation time can be ignored since it will affect the real-time of localization in the online stage. In the online stage of the proposal, according to Equations (13)–(16), the computation time complexity of localization estimation is O(N).

In total, whatever the number of fingerprints in the competitors is (in the range of 1 to 20), the performance of the proposal is always superior to that of the competitors with a similiar computation time cost; thus, the validity of proposal can be testified.

Moreover, in order to highlight the effectiveness of the proposed HATRFLA, in our experiments, we used the simplest equipment under NLOS communication conditions to increase the difficulty of localization. To be specific, the CSI information was transmitted from a transmitter equipped with one antenna to a receiver equipped with one antenna was used in our experiment wherein the limited bandwidth of the one available channel was 20 MHz and the number of APs was 1. In fact, as indicated by the experimental results in Section 3.1, the proposed approach can be extended to scenarios with multi-antennas, multi-APs, and/or multi-channels to further improve the localization accuracy. Given that this is not the focus of this paper, it is not described here.

## 4. Conclusions

We conducted experiments to evaluate the influence of factors on TR localization, and we found that the localization performance deteriorated sharply when the bandwidth was limited, e.g., 20 MHz. To improve the WiFi localization accuracy, we proposed a high accuracy TR based fingerprinting localization approach, i.e., HATRFLA, in this paper. In the offline stage of this approach, to minimize fingerprint database storage space and, meanwhile, to pick the fingerprints containing valid, location-specified information, a density-based spatial clustering algorithm is employed to adaptively obtain the optimal fingerprints for each location. In the online stage, the HATRFLA makes full use of the two unique location-specified signatures extracted from CSI to achieve better accuracy than existing TR localization approaches without requiring more fingerprint data storage space. The experimental results show that the proposal achieves high localization accuracy using a finite number of fingerprints per location with 20 MHz bandwidth, outperforming the existing TR localization approaches.
